# The Preliminary Education of Medical Students

**Published:** 1875-07

**Authors:** 


### Editorial.

### The Preliminary Education of Medical Students.

We clip from the *Buffalo Express* of July 19th, the following in reference to the preliminary qualifications of medical students:

MEDICAL PROSCRIPTION.—If we may judge from the statements of one of their own guild, "The Lake Erie Medical Society" is disposed to "play it" with a pretty high hand. On the authority of one of the most prominent members of the Society, they propose who may and *may not study medicine* with any "regular" practitioner within their limits. The proposition was introduced at the meeting of the Society at Mayville last week and adopted; it professed to have originated with the *State* Medical Society. If so, it is to be understood that the whole allopathic connection in this State propose to establish a sort of medical trades union and bar out apprentices, and to put a monopoly on medical knowledge. We are curious to know if such a proscription is sanctioned by the respectable and liberal gentlemen of that guild and we therefore forbear further comment, only presenting the statement on what we base our present comments:

*Medical Censors.*—The Lake Erie Medical Society met Tuesday at the Fox house in Mayville. * * * * Nothing occurred to mar the family gathering till Dr. Strong, of Westfield, offered a resolution to form a board of censors, their duty being to examine ever applicant for admission into any office in the limits of this Society to study medicine. The doctor stated the matter came to him as a request from the State Medical Society, the object being to raise the standard of the medical profession of the State. This worthy motive was ably urged in the affirmative by several of the members. The negative was taken by myself, and maintained that quackery flourished through ignorance among the people, and that to remove the evil the people must be better educated in the medical sciences, contending for its introduction into common schools, and that every avenue be opened to the people to acquire medical knowledge, that every doctor was in duty bound to instruct his patrons habitually, and further, to receive any and all students, male and female, that may apply at his office, that he may in his own good pleasure feel willing to instruct. That every college shall open its doors wide to all who may wish to attend, and that the graduating standard be placed as high as the country demands. That the resolution invaded the sacred rights of my own office, to which I could under no circumstances ever consent. The resolution passed, and I first resigned the position of censor to which I had been elected, and then demanded that my name be stricken from the rolls of the Society. To all the members of the Society I have but the kindest feelings, but my manhood and sense of duty compelled me to this course. I now call upon the Society and all others to unite against this effort to place our medical schools at the mercy of rings and personal ambition.

G. W. WHITNEY, M. D.

Jamestown, July 13, 1875.

In times past the secular press have seemed to take particular pleasure in holding up to public gaze the lack of culture and intelligence which exists to a deplorable extent in the ranks of a so called learned profession, and we confess that we are surprised at the stand taken by the *Express* and its correspondent in this case.

The editor of the *Express* could not have taken much pains to enquire into the merits of the case or he would not have entitled the attempt to raise the standard of intelligence requisite to entrance upon the study of medicine, an act of *medical proscription.* The members of the medical profession do not seek to bar out any worthy applicant from their ranks, they welcome all who come with a worthy motive and an intelligence capable of appreciating the difficult and important problems which the calling presents. For such there is room and to spare, for all others the ranks are crowded, and each additional acquisition only serves as a dead weight which drags down the entire body and cause the term *medical education* to become a by-word and reproach among men.

The object of the resolution, which originated in the American Medical Association, and has since been adopted by the New York State Medical Society and several county and district medical associations, is to provide that those who are about to enter upon the study of medicine shall be required to present some evidence that they possess an amount of preliminary education which shall render them capable of understanding the study which they have undertaken.

In no country in the world is the standard of entrance so low as it is in America, and the remedy proposed is far beneath that required for entrance in any of the Continental schools.

Surely the editor of the *Express* would expect that a medical student would be required to know as much at least as an ordinary type-setter in the printing office of his paper. Dr. Whitney who objects so strongly against having his rights invaded would not, we hope, receive into his office as a student of medicine any one whose literary qualifications were below those demanded in the resolution introduced by Dr. Strong.

The doctor objects to having his rights usurped, he wishes to be his own judge of the qualifications of his student. If Dr. Whitney was alone concerned we might be inclined to allow his claim, as he would probably make a proper selection, but when so many different views are to be met some established standard and authority must be fixed, and as long as the colleges do not require an entrance examination the medical societies must see to it that the material sent them is of the right kind. In this the members of the profession seek to exercise no proscription, they have learned by the experience of the past, many of them by a personal one, that a substantial preliminary education is a necessary foundation upon which to build the superstructure of medical learning. They ask therefore of the young men whom they are about to receive as students, both in justice to themselves and to their pupils, that they come to them suitably prepared to receive instruction.

The examination of young men commencing the study of medicine by the censors of the county society is one step in advance, and as such it should be greeted with joy; but it possesses many defects and disadvantages. Some of these time and experience will rectify. The only true solution of the problem remains however with our medical colleges. The time is coming, we hope, when they will take this matter in hand and require applicants for admission to their instruction to pass a suitable examimation. This cannot yet be undertaken, for it requires concert of action on the part of all of the medical schools. Under our present system it will not do for our poorer and weaker schools to demand this, for their students would immediately flock to some school which had no such requirement. But we hope that the time is coming soon when all of our best schools will find it for their interest to undertake this step. When this is done the next step, namely the elevation of the standard for graduation will be natural and easy, for the greater intelligence of the candidates for graduation will cause this to be expected. Dr. Whitney's asservation that the people need educating in the medical sciences is a correct one, but his deduction that hence all who apply to a doctor for instruction should be received is not a natural one. We need to educate the people but it may be done as much by example as by precept. Until they learn to recognize a material difference between the votaries of medical science, and the charlatans who flaunt the high sounding titles and pretentious dogmas of quackery in their faces it will be a bootless task to attempt to instruct them in any of the principles of medical science. They certainly will have little respect for a science which will receive students direct from the plow or bench, *whose mental training if any, is very defficient indeed, and after three years desultory and unintelligent reading, dub him a doctor and send him forth to practice upon mankind.* A diploma in any of the other learned professions means more than that the holder has gone through with a prescribed course of study in his profession, it means that he is possessed of a liberal education outside of the professional lines, and the holder is respected accordingly. In the medical profession it is far different, and yet no other calling makes greater demand upon the intelligence and mental training of its followers. It has been in time past the opprobrium of the American medical profession that its ranks were open to all comers, and those who at this time seek to take away the reproach should not be accused of proscription. Literary colleges demand a preliminary examination as a requisite to entrance, and the medical profession have a right, and should in justice to themselves and those who propose to enter the ranks, require that the student shall by previous mental training have fittted himself to undertake in an intelligent manner the prescribed course of study. If the medical profession desire to be entitled to the term respectable and liberal, which the editor of the *Express* so kindly applies to them, they must make themselves worthy of it. It is only by showing the world that they are an educated and liberal body in every sense that they can gain respect, and it is only by starting right in the pursuit of their profession that they can attain the desired end. This being the case, the press and every member of the profession should aid in every way toward accomplishing an end so greatly to be desired. B.

